# Pd/δ-MnO_2_ nanoflower arrays cordierite monolithic catalyst toward toluene and *o*-xylene combustion

**DOI:** 10.3389/fchem.2022.978428

**Published:** 2022-10-13

**Authors:** Yongfeng Li, Qianyan Liao, Weizhao Ling, Fan Ye, Fangfang Liu, Xipeng Zhang, Jiajun He, Gao Cheng

**Affiliations:** ^1^ Key Laboratory of Clean Chemistry Technology of Guangdong Regular Higher Education Institutions, School of Chemical Engineering and Light Industry, Guangdong University of Technology, Guangzhou, China; ^2^ Guangdong Provincial Key Laboratory of Plant Resources Biorefinery, Guangdong University of Technology, Guangzhou, China

**Keywords:** Pd nanoparticles, δ-MnO_2_ nanoarrays, catalytic combustion, toluene, *o*-xylene, synergy effect, surface-absorbed oxygen, particle size

## Abstract

Exploring high-efficiency and stable monolithic structured catalysts is vital for catalytic combustion of volatile organic compounds. Herein, we prepared a series of Pd/δ-MnO_2_ nanoflower arrays monolithic integrated catalysts (0.01–0.07 wt% theoretical Pd loading) *via* the hydrothermal growth of δ-MnO_2_ nanoflowers onto the honeycomb cordierite, which subsequently served as the carrier for loading the Pd nanoparticles (NPs) through the electroless plating route. Moreover, we characterized the resulting monolithic integrated catalysts in detail and evaluated their catalytic activities for toluene combustion, in comparison to the controlled samples including only Pd NPs loading and the δ-MnO_2_ nanoflower arrays. Amongst all the monolithic samples, the Pd/δ-MnO_2_ nanoflower arrays monolithic catalyst with 0.05 wt% theoretical Pd loading delivered the best catalytic performance, reaching 90% toluene conversion at 221°C at a gas hourly space velocity (GHSV) of 10,000 h^−1^. Moreover, this sample displayed superior catalytic activity for *o*-xylene combustion under a GHSV of 10,000 h^−1^. The monolithic sample with optimal catalytic activity also displayed excellent catalytic stability after 30 h constant reaction at 210 and 221°C.

## Introduction

Nowadays, extensive attention is being paid to the effective reduction of gaseous pollutants, such as NO_x_, CO_2_, VOCs, and so forth (Yao et al., 2015; Yao et al., 2017; Guo et al., 2021; Ji et al., 2021; Li et al., 2021; Zhang et al., 2022). Of these gases, Volatile organic compounds (VOCs) are major precursors of air pollutants (e.g., PM_2.5_ and O_3_), and some VOCs (e.g., toluene and acetone) even directly endanger human health and atmospheric environment (Gan et al., 2019; Fu et al., 2022). Thus, reducing the VOCs emission is extremely critical for the sustainable development of human society and ecological environment (McDonald et al., 2018). To reduce VOCs emissions, several techniques have been well exploited, such as adsorption, thermal incineration, and catalytic combustion. Amongst them, catalytic combustion technique is now deemed as one promising pathway in VOCs elimination filed for its known effectiveness and economy (He et al., 2019). Developing high-efficiency catalysts is the core of catalytic combustion technology, in which noble metal (e.g., Pd and Pt) based catalysts are quite attractive because of their outstanding catalytic performance (Gan et al., 2019; Bi et al., 2020; Song et al., 2020). However, the large-scale application of noble metal-based catalysts is still restricted by their high cost because of large noble metals consumption and easy poisoning. Moreover, due to the high surface energy, the noble metal NPs are easy to be aggregated into larger particles during the preparation process or the on-going catalytic reaction, leading to a decrease in the catalytic activity (Han et al., 2019; Bi et al., 2020). Consequently, it is urgently desirable to deposit the noble metal onto a carrier to achieve a supported noble metal NPs with highly dispersion, and thereby the aggregation of noble metal NPs and high consumption of noble metals can be alleviated (Lou et al., 2020a).

Monolithic integrated catalytic reactors, typically composed of a monolithic structured substrate, a washcoat, and an active component, have been extensively used in various gas-solid catalytic reactions owing to their unique advantages such as efficient mass transport, great mechanical strength, and easy large-scale production (Govender and Friedrich, 2017). However, the washcoat-based catalysts lack the well-defined structure and morphology, which leads to the low internal mass-transfer efficiency and compromised catalytic performance. To circumvent these issues, many efforts have focused on direct growth of nanostructures onto monolithic substrate without a supporting washcoat (Ren et al., 2015; Lu et al., 2021). Such monoliths coated with nanostructures were endowed with strong adhesion, uniform morphology, and rich active sites, thus exhibiting tunable catalytic activities toward various oxidation reactions. Previously, our group employed an electroless plating method to directly deposit noble metal (Pd and Pt) NPs onto the monolithic substrate, showing great potential as efficient catalysts for toluene combustion (Li et al., 2012; Li et al., 2017a). However, the size of noble metal NPs was too large (≥75 nm), which compromised the utilization efficiency of noble metal and decreased the active sites of noble metal NPs. At present, the monoliths coated with well-organized metal oxide nanoarrays are being promising catalysts for VOCs oxidation (Tang et al., 2017; Zhang et al., 2018; Zhao et al., 2020). What’s more, these metal oxide nanoarrays onto the monolithic substrate can effectively enhance the substrate’s surface area, contributing to the dispersion and size reduction of noble metal NPs. In this respect, significantly boosted catalytic activity is expected to be realized in such a composite type of noble metal/metal oxide monolithic integrated catalysts (Fu et al., 2022).

**FIGURE 1:**
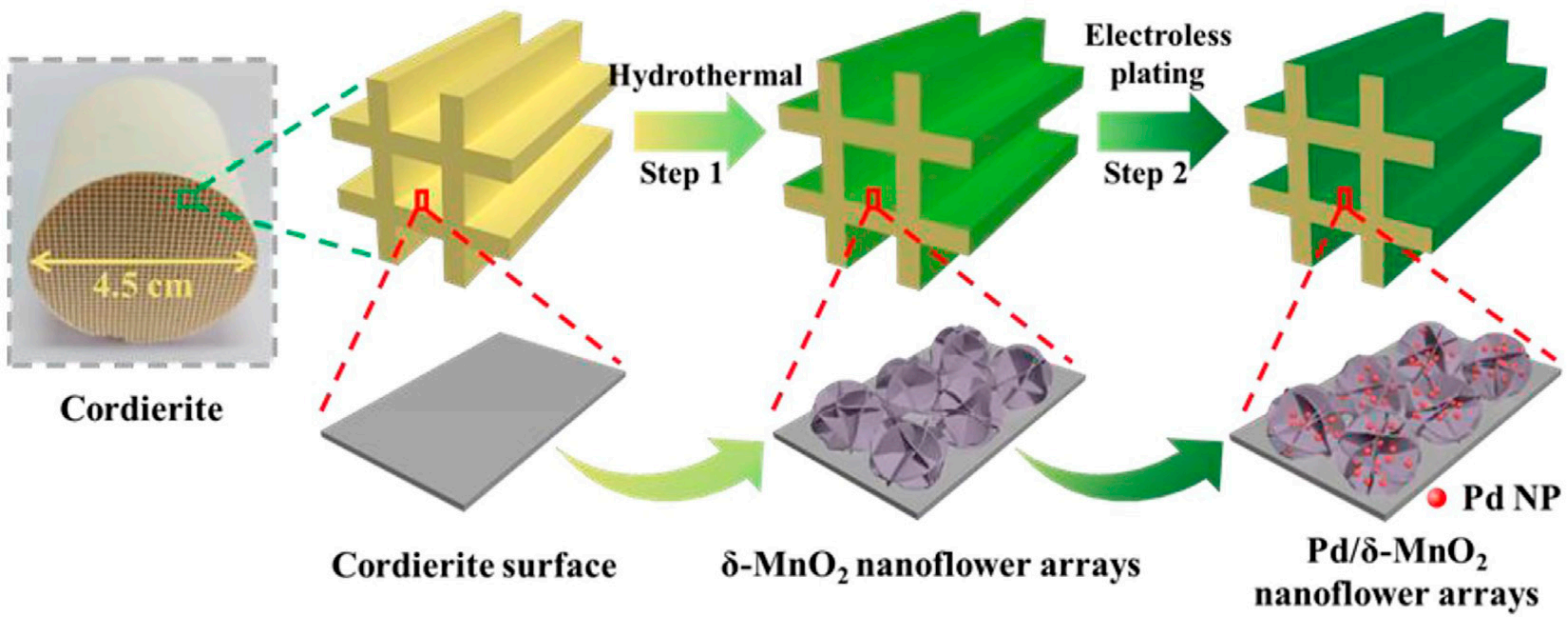
Schematic illustration of synthesis of Pd/δ-MnO_2_ nanoflower arrays.

**FIGURE 2:**
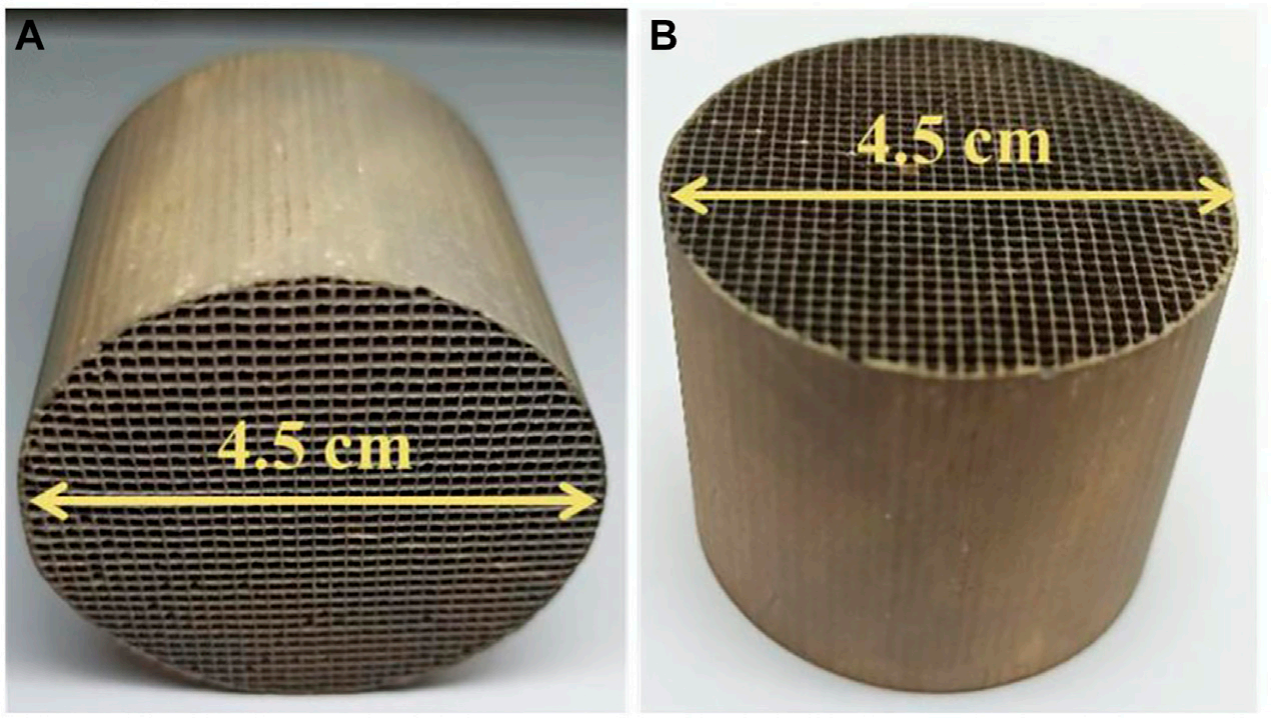
**(A, B)** Photos of Pd/δ-MnO_2_ nanoflower arrays monolithic sample.

Herein, by using a two-step synthetic route (Figure 1), δ-MnO_2_ nanoflower arrays supported Pd NPs have been successfully integrated onto honeycomb cordierite substrate. Firstly, we readily constructed uniform δ-MnO_2_ nanoflower arrays on the cordierite surface through a hydrothermal method. Next, the electroless plating method was utilized to deposit Pd NPs on the surface of δ-MnO_2_ nanoflower. The photos of the resulting Pd/δ-MnO_2_ nanoflower arrays monolithic sample are given in Figure 2. After loading onto the nanoflower, the Pd NPs exhibited both better dispersion and smaller particle size than those directly depositing onto the cordierite substrate. As expected, the resulting Pd/δ-MnO_2_ nanoflower arrays monolithic catalyst was highly active for toluene and *o*-xylene combustion, especially for the Pd/δ-MnO_2_ nanoflower arrays monolithic sample with ultralow theoretical Pd loading (0.05 wt%). This optimal monolithic catalyst also exhibited an excellent long-term durability.

## Experimental

### Materials

Sodium hydroxide (NaOH), ethylenediamine tetracetic acid disodium salt (C_10_H_14_N_2_O_8_Na_2_·2H_2_O, EDTA), potassium permanganate (KMnO_4_), ammonium Chloride (NH_4_Cl), sodium hypophosphite (NaH_2_PO_2_·H_2_O), and ammonia water (25%) were bought from Guangzhou Chemical Reagent Co. Ltd. (Guangzhou, China). Palladium (Ⅱ) Chloride (PdCl_2_) was purchased from Shaoyuan Co. Ltd. (Shanghai, China). Sodium borohydride (NaBH_4_) was obtained from Aladdin Bio-Chem Technology Co. Ltd. (Shanghai, China). All the above reagents were used directly without purification. Commercial honeycomb cordierite was bought from Huanya Chemical Packing Co. Ltd. (Pingxiang, China). Before growth of catalysts, a commercial honeycomb cordierite (5 mm × 5 mm × 20 mm) was immersed and cleaned in the dilute nitric acid for ∼30 min, and rinsed with deionized water. Then it was dried at 80 °C overnight for later use.

### Preparation of monolithic catalysts

#### Growth of δ-MnO_2_ nanoflower arrays

Uniform δ-MnO_2_ nanoflower arrays were loaded onto the cordierite substrate by a low-temperature hydrothermal route. Typically, 15 ml of CH_3_COOH solution (0.1 M) was poured into a Teflon liner (25 ml), in which 1.5 mmol KMnO_4_ was dissolved to form a mixed solution under continuous stirring for ∼30 min. Then the clean cordierite substrate was put vertically on the bottom of liner. After hydrothermally maintained at 100°C for 12 h, the modified cordierite was carefully cleaned with deionized water and ethanol, then dried at 80°C for further use. The as-obtained monolithic sample was denoted as δ-MnO_2_-NFA.

### Preparation of Pd/δ-MnO_2_ nanoflower arrays

A series of *x* wt% Pd/δ-MnO_2_ nanoflower arrays monolithic samples (*x*: theoretical mass ratio of Pd to δ-MnO_2_-NFA, *x* = 0.01, 0.02, 0.03, 0.05, and 0.07 wt%) were prepared using the electroless plating route (Li et al., 2012; Li et al., 2017a). 1) Activization. The as-prepared δ-MnO_2_-NFA sample was immersed in 50 ml mixed solution for 15 min (PdCl_2_: 0.05 g/L, EDTA: 0.4 g/L, pH = 9–11). 2) Chemical plating of Pd. Then the activated δ-MnO_2_-NFA sample was put in the 50 ml platinum plating solution at 45–55°C for 4 h (PdCl_2_: 0.0005–0.003 g/L, NaBH_4_: 0.1 g/L, NH_4_Cl: 13.5 g/L, NaH_2_PO_2_: 5 g/L, NH_4_OH: 80 ml/L). The usage of PdCl_2_ was based on the theoretical Pd loading. 3) Drying. After being cleaned with deionized water, the resulting monolithic sample was dried at 80°C overnight. The *x* wt% Pd/δ-MnO_2_ nanoflower arrays monolithic sample was denoted as *x*Pd/δ-MnO_2_-NFA. The loading mass of Pd/δ-MnO_2_ nanoflower arrays is ∼1 mg.

### Deposition of Pd nanoparticles onto cordierite

For comparison, the pure cordierite substrate supported Pd nanoparticles with 0.05 wt% theoretical loading amount was prepared by the electroless plating route under the same procedures described above (part of Preparation of Pd/δ-MnO_2_ nanoflower arrays). The as-prepared 0.05 wt% Pd nanoparticles monolithic sample was denoted as 0.05Pd-NP.

### Characterization

Structure identification of the monolithic samples was carried out by X-ray diffractometer (XRD, Bruker D8 ADVANC) using Cu K*α* irradiation. Their morphologies and microstructures were investigated by field emission scanning electron microscope (FESEM, Hitach, SU8220) and transmission electron microscope (TEM, FEI Talos F200S). The average particle size of Pd NPs was obtained by counting at least 100 single Pd NPs from the FESEM image. Brunauer-Emmett-Teller (BET) surface areas of the monolithic samples were acquired using Quantachrome Autosorb iQ instrument at −196°C. Inductively coupled plasma mass spectroscopy (ICP-MS) technique was applied to acquire the actual Pd amount in the monolithic samples containing Pd-based species. Surface compositions and their binding energies of the samples were analyzed using X-ray photoelectron spectroscopy (XPS, Escalab 250Xi) equipped with Al K*α* source.

### Catalyst activity test

The catalytic activity test for toluene or *o*-xylene combustion was performed in a quartz tube (*Φ* = 8 mm). Typically, the as-prepared monolithic samples with 16 channels (5 mm × 5 mm × 10 mm) were utilized to evaluate the catalytic performance. The gaseous reactant was consisted of 250 ppm toluene, O_2_ (20%) and N_2_ (balance), and the total flow rate is 51 ml/min. Hence the GHSV is 10,000 h^−1^. Catalytic oxidation measurement was conducted in a temperature range of 110–300°C using a heating furnace. The concentrations of outlet gases were monitored in real time by a gas chromatograph (Agilent 6820) with flame ionization detector (FID), and no other products were found except CO_2_ and H_2_O. Therefore, the toluene or *o*-xylene conversion can be calculated by the consumption of toluene or *o*-xylene. The values of activation energies (*E*_a_) were computed based on the equation (ln *k* = −*E*_a_/*RT +* ln *A*) derived from the Arrhenius equation, where *k* is the rate constant (s^−1^) and *A* is the pre-exponential factor. The *E*_a_ values were calculated with toluene conversion below 15%.

## Results and discussion

### Catalytic performance

The effect of Pd loading (from 0.01 to 0.07 wt%) on the catalytic activities for the Pd/δ-MnO_2_-NFA samples was firstly investigated (Supplementary Figure S1A). For comparison, *T*_10_, *T*_50_, and *T*_90_ (temperatures at 10%, 50%, and 90% toluene conversion, respectively) are used to evaluate the catalytic performance of Pd/δ-MnO_2_-NFA samples (Supplementary Figure S1B). With the increased dosage of Pd^2+^ precursor, the catalytic activities of Pd/δ-MnO_2_-NFA samples are significantly promoted. Notably, the 0.07Pd/δ-MnO_2_-NFA sample presents the highest catalytic performance: the *T*_10_, *T*_50_, and *T*_90_ are 152, 203, and 219°C, respectively. Moreover, the *T*_10_, *T*_50_, and *T*_90_ of 0.05Pd/δ-MnO_2_-NFA (149, 206, and 221°C) are nearly close to those of 0.07Pd/δ-MnO_2_-NFA. Considering both Pd^2+^ dosage and catalytic activity, Pd/δ-MnO_2_-NFA with 0.05 wt% Pd loading is the preferred sample for further investigation.

**FIGURE 3:**
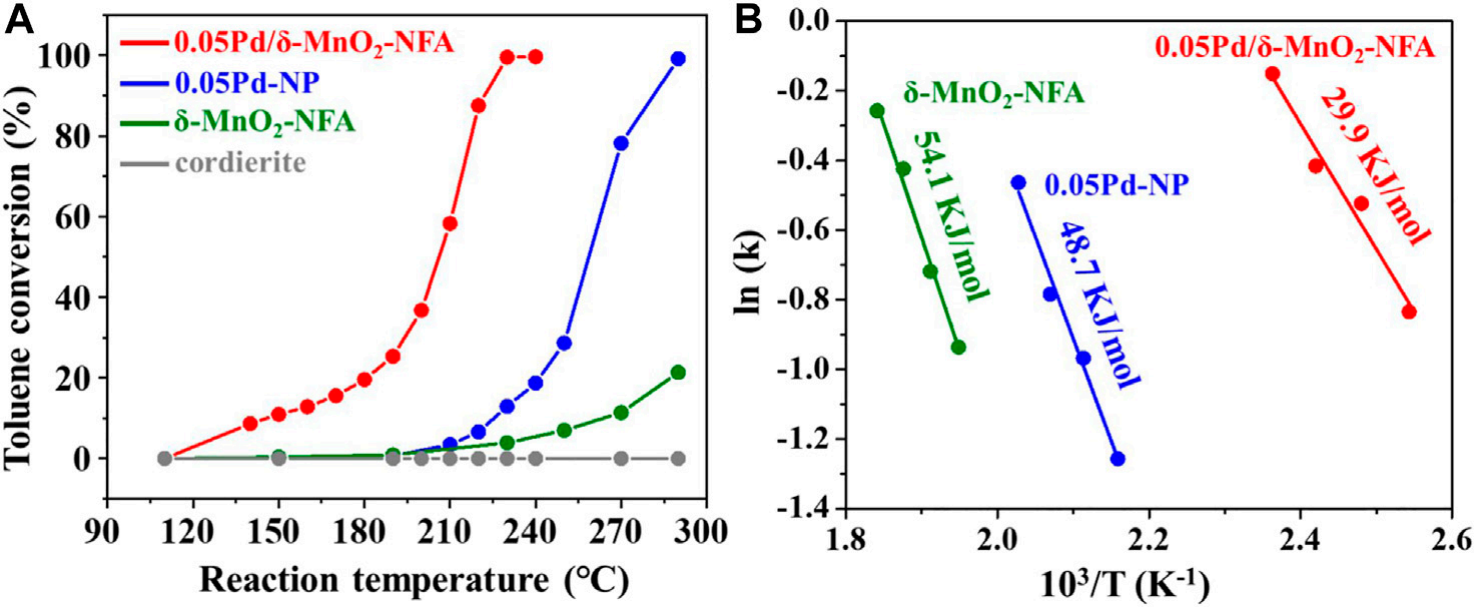
**(A)** Toluene conversion over all the monolithic samples; **(B)** Arrhenius plots (toluene conversion below 15%) over 0.05Pd/δ-MnO_2_-NFA, 0.05Pd-NP, and δ-MnO_2_-NFA samples.

Figure 3A and Table 1 present the catalytic activities of toluene combustion over 0.05Pd/δ-MnO_2_-NFA, 0.05Pd-NP, δ-MnO_2_-NFA, and cordierite substrate. Obviously, the cordierite substrate exhibits no reactivity for toluene oxidation in the temperature range of 110–300°C. For the δ-MnO_2_-NFA sample, the toluene conversion is pretty low and only ∼20% toluene conversion is detected at 290°C. With only Pd loading, the 0.05Pd-NP sample exhibits a dramatically superior activity compared to the δ-MnO_2_-NFA sample, with *T*_10_, *T*_50_, and *T*_90_ at 226, 258, and 281°C, respectively. Depositing Pd NPs onto the δ-MnO_2_-NFA surface further improves the catalytic performance toward toluene combustion. The remarkable toluene oxidation activity in the 0.05Pd/δ-MnO_2_-NFA sample might be due to the strong synergy between Pd NPs and δ-MnO_2_-NFA support (Liu et al., 2017). The catalytic performance (e.g., value of *T*_90_) over the 0.05Pd/δ-MnO_2_-NFA sample is further compared with other Pd-based monolithic integrated catalysts reported in the previous studies (Kim et al., 2012; Liu et al., 2020; Liu et al., 2021). For example, under the GHSV of 10,000 h^−1^, the 0.05Pd/δ-MnO_2_-NFA catalyst shows better toluene oxidation activity (*T*_90_ = 221°C) than the 0.20 wt% Pd-AlOOH/Al (*T*_90_ = 233°C) and 0.05 wt% Pd-AlOOH/Al-EDTA catalysts (*T*_90_ = 230°C) (Liu et al., 2020; Liu et al., 2021). When the GHSV rises to 40,000 h^−1^, the 0.05Pd/δ-MnO_2_-NFA catalyst also exhibits superior catalytic activity for toluene combustion compared to the 0.1 wt% Pd/cordierite honeycomb catalyst (Kim et al., 2012).

**TABLE 1:** Values of *T*_10_, *T*_50_, *T*_90_, and *E*_a_ for toluene oxidation.

Sample	*T*_10_ (°C)	*T*_50_ (°C)	*T*_90_ (°C)	*E*_a_ (kJ/mol)
0.05Pd/δ-MnO_2_-NFA	149	206	221	29.9
0.05Pd-NP	226	258	281	48.7
δ-MnO_2_-NFA	270	—	—	54.1

**FIGURE 4:**
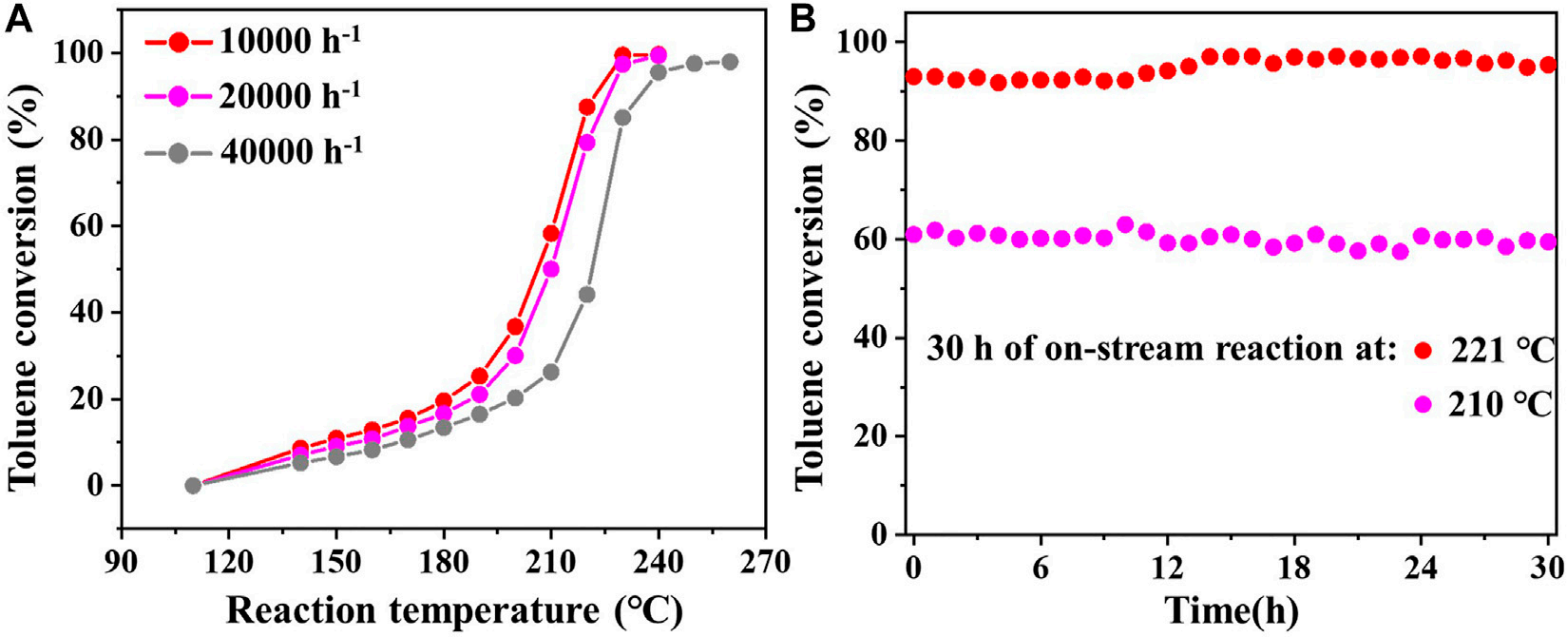
**(A)** Effect of GHSV on the toluene conversion for the 0.05Pd/δ-MnO_2_-NFA sample; **(B)** catalytic stability of toluene and *o*-xylene combustion over 0.05Pd/δ-MnO_2_-NFA at a GHSV of 10,000 h^−1^.

Figure 3B displays the Arrhenius plots for toluene combustion over 0.05Pd/δ-MnO_2_-NFA, 0.05Pd-NP, and δ-MnO_2_-NFA. The *E*_a_ was computed by the linear-fitting plots (ln *k* vs. 1/*T*). As summarized in Table 1, the calculated *E*_a_ values follow the order: δ-MnO_2_-NFA (54.1 kJ/mol) > 0.05Pd-NP (48.7 kJ/mol) > 0.05Pd/δ-MnO_2_-NFA (29.9 kJ/mol), showing the reverse order when comparing to the sequence of their catalytic performance. The lower *E*_a_ value means the easier toluene combustion (Ren et al., 2019). Thus, the lowest *E*_a_ value of 0.05Pd/δ-MnO_2_-NFA among the three samples further confirms its best catalytic efficiency for toluene combustion. The effect of GHSV on the catalytic performance of 0.05Pd/δ-MnO_2_-NFA is given in Figure 4A. The toluene conversion declines with an increasing GHSV from 10,000 to 40,000 h^−1^ because of the shortened contact time between the catalyst surface and the reactants (Fu et al., 2022). Subsequently, we investigated the catalytic stability of 0.05Pd/δ-MnO_2_-NFA sample by measuring the toluene conversion within 30 h of on-stream reaction at 210 (*T*_60_) and 221°C (*T*_90_). Impressively, the toluene conversion for the 0.05Pd/δ-MnO_2_-NFA sample is not obviously changed (Figure 4B), demonstrating its excellent catalytic stability for toluene combustion.

Moreover, *o*-xylene catalytic combustion was conducted for further comparing the catalytic activities of 0.05Pd/δ-MnO_2_-NFA, 0.05Pd-NP, δ-MnO_2_-NFA, and cordierite substrate (Supplementary Figure S2). As expected, the activity trend of *o*-xylene catalytic combustion is perfectly in line with that of toluene combustion. As displayed in Table 2, the 0.05Pd/δ-MnO_2_-NFA is more active than the 0.05Pd-NP and δ-MnO_2_-NFA. According to the above catalytic activity results, the 0.05Pd/δ-MnO_2_-NFA exhibits superior catalytic performance and admirable stability, revealing 0.05Pd/δ-MnO_2_-NFA as an ideal catalyst for VOCs elimination.

**TABLE 2:** Values of *T*_10_, *T*_50_, and *T*_90_ for *o*-xylene oxidation.

Sample	*T*_10_ (°C)	*T*_50_ (°C)	*T*_90_ (°C)
0.05Pd/δ-MnO_2_-NFA	183	196	206
0.05Pd-NP	205	239	260
δ-MnO_2_-NFA	290	—	—

### Structure and morphology characterization

**FIGURE 5:**
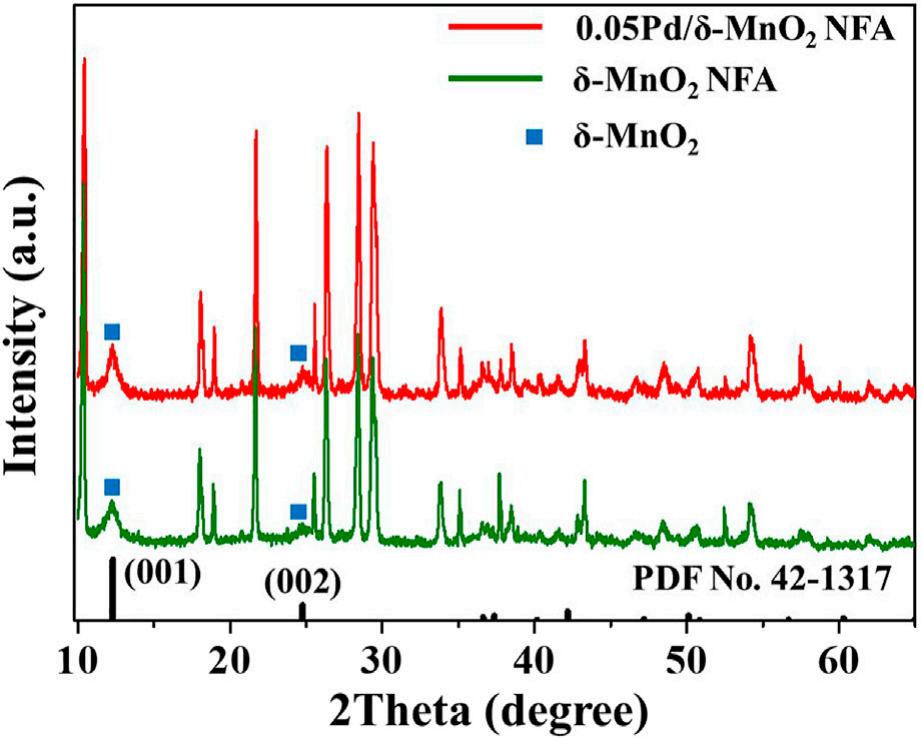
XRD patterns of 0.05Pd/δ-MnO_2_-NFA and δ-MnO_2_-NFA samples.

XRD technology was conducted to determine the crystalline structure of the components loaded onto the cordierite. The XRD patterns of 0.05Pd/δ-MnO_2_-NFA, 0.05Pd-NP, δ-MnO_2_-NFA, and cordierite substrate are given in Figure 5 and Supplementary Figure S3. Obviously, the strong diffraction peaks indexed to cordierite (2MgO·2Al_2_O_3_·5SiO_2_, PDF No. 12-0303) are observed for the 0.05Pd/δ-MnO_2_-NFA, δ-MnO_2_-NFA, and 0.05Pd-NP samples. Note that two distinct diffraction peaks at 12.4° and 24.8° appear in the 0.05Pd/δ-MnO_2_-NFA and δ-MnO_2_-NFA (Figure 5), which are well assigned to the (001) and (002) planes of monoclinic δ-MnO_2_ (PDF No. 42-1317). Moreover, no diffraction peaks associated with Pd-based species such as Pd or PdO can be detected in the 0.05Pd/δ-MnO_2_-NFA and 0.05Pd-NP, which is likely due to the low loading of Pd-based components (Xu et al., 2017).

**FIGURE 6:**
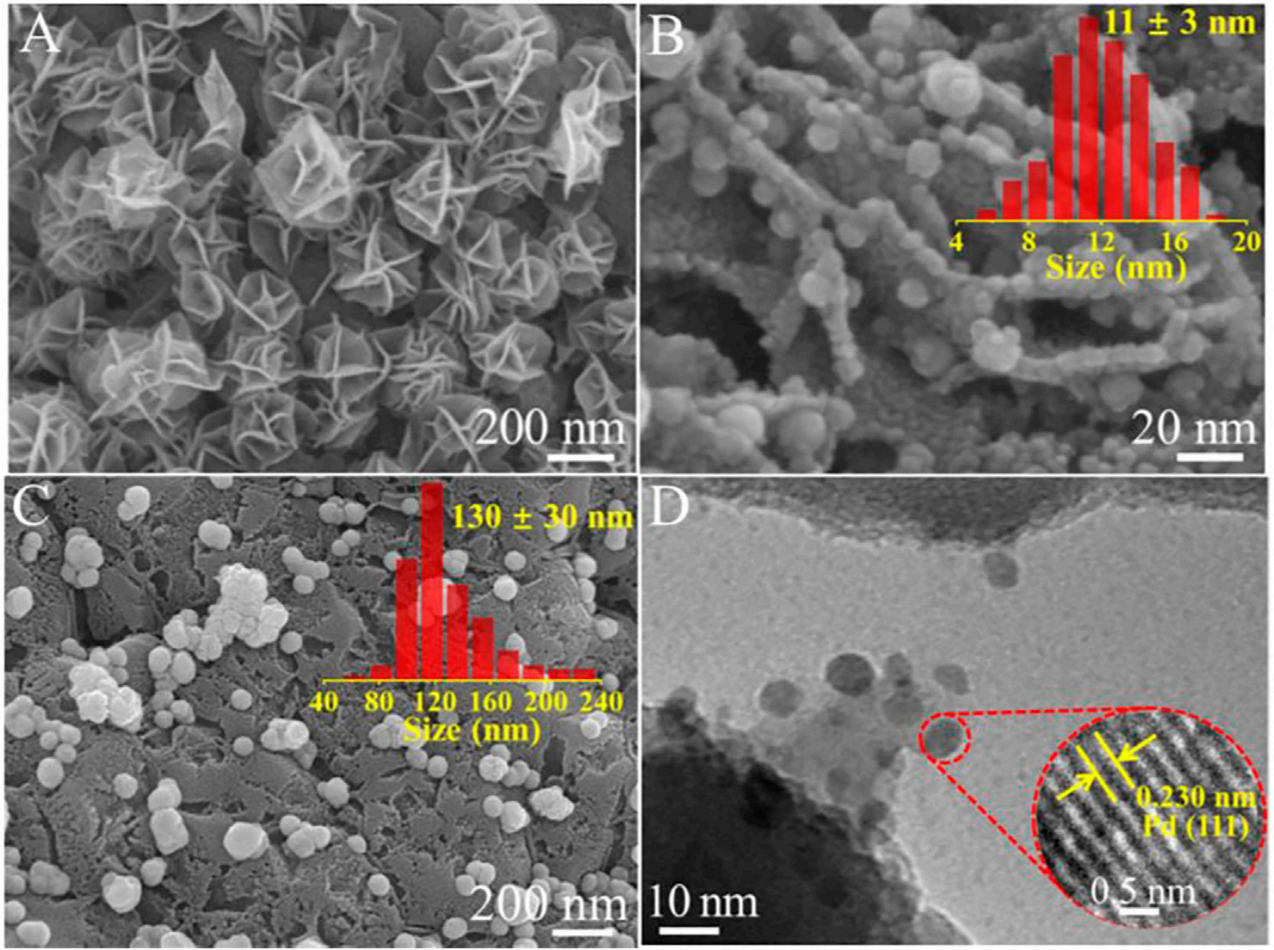
**(A)** FESEM image of δ-MnO_2_-NFA; **(B)** FESEM image of 0.05Pd/δ-MnO_2_-NFA (inset: size distribution of Pd NPs); **(C)** FESEM image of 0.05Pd-NP (inset: size distribution of Pd NPs); **(D)** TEM image of 0.05Pd/δ-MnO_2_-NFA (inset: HRTEM image of Pd nanoparticle).

FESEM was applied for comparing the surface morphologies of pure cordierite substrate, δ-MnO_2_-NFA, 0.05Pd/δ-MnO_2_-NFA, and 0.05Pd-NP. As illustrated in Supplementary Figure S4, the pure cordierite exhibits a relatively smooth and clean surface. Owing to the successful growth of δ-MnO_2_ nanoarrays on the cordierite surface, the obvious rough surface can be observed for the δ-MnO_2_-NFA sample (Supplementary Figure S5A). The δ-MnO_2_ nanoarrays exhibit the flower-like morphology (Supplementary Figure S5B). Supplementary Figure S5C indicates that the δ-MnO_2_ nanoflower arrays is ∼200 nm in height. The closer observation (Figure 6A) reveals that each δ-MnO_2_ nanoflower (250–400 nm in diameter) is composed of many nanosheets with a thickness of ∼5 nm, offering a uniformly hierarchical and open structure for the deposition of Pd NPs. Figure 6B presents a magnified FESEM image of the 0.05Pd/δ-MnO_2_-NFA sample. Clearly, the Pd NPs with an average size of 11 ± 3 nm are highly dispersed over the surface of δ-MnO_2_ nanoflowers. However, when using cordierite substrate as the support, distinct change in size for the Pd NPs is observed in the 0.05Pd-NP sample (Figure 6C). It is found that the Pd NPs with a larger size of 130 ± 30 nm are randomly deposited on the cordierite surface (inset of Figure 6C). The FESEM results verify that the δ-MnO_2_ nanoflower arrays can serve as the ideal support to anchor the Pd NPs and efficiently prevent their further aggregation during the synthetic process of electroless plating. Therefore, the size of deposited Pd NPs can be well adjusted by virtue of the steric hindrance effect by the δ-MnO_2_ nanoflowers (Zheng et al., 2021; Shi et al., 2022).

In order to further analyze the microstructure of the Pd/δ-MnO_2_ nanoflower arrays, a small quantity of gray powders was scraped off from the surface of 0.05Pd/δ-MnO_2_-NFA sample and collected for TEM characterization. Figure 6D reveals that Pd NPs are well anchored onto the δ-MnO_2_ nanosheet with no aggregation, which is in accord with the FESEM result. The clear lattice spacing of 0.230 nm in the HRTEM image (inset of Figure 6D) well matches the (111) crystal plane of Pd. Supplementary Figure S6 presents the N_2_ adsorption-desorption isotherms of the δ-MnO_2_-NFA and pure cordierite substrate. These two monoclinic samples exhibit the similar isotherm, both of which are assigned to type II curve with H3 hysteresis ring (Thommes et al., 2015). After coating the δ-MnO_2_ nanoflowers on the cordierite surface, the BET surface area of δ-MnO_2_-NFA sample is enlarged (11.37 m^2^/g), which is higher than that of pure cordierite substrate (3.21 m^2^/g). Additionally, the ICP-MS result confirms that the actual Pd loading amount of 0.05Pd/δ-MnO_2_-NFA (0.030 wt%) is more than that of 0.05Pd/NP (0.021 wt%). Compared with the pure cordierite substrate, the δ-MnO_2_-NFA sample with increased surface area is more beneficial for the dispersion of Pd NPs with smaller size, thus improving the utilization efficiency of Pd^2+^ precursor (Fu et al., 2022).

**FIGURE 7:**
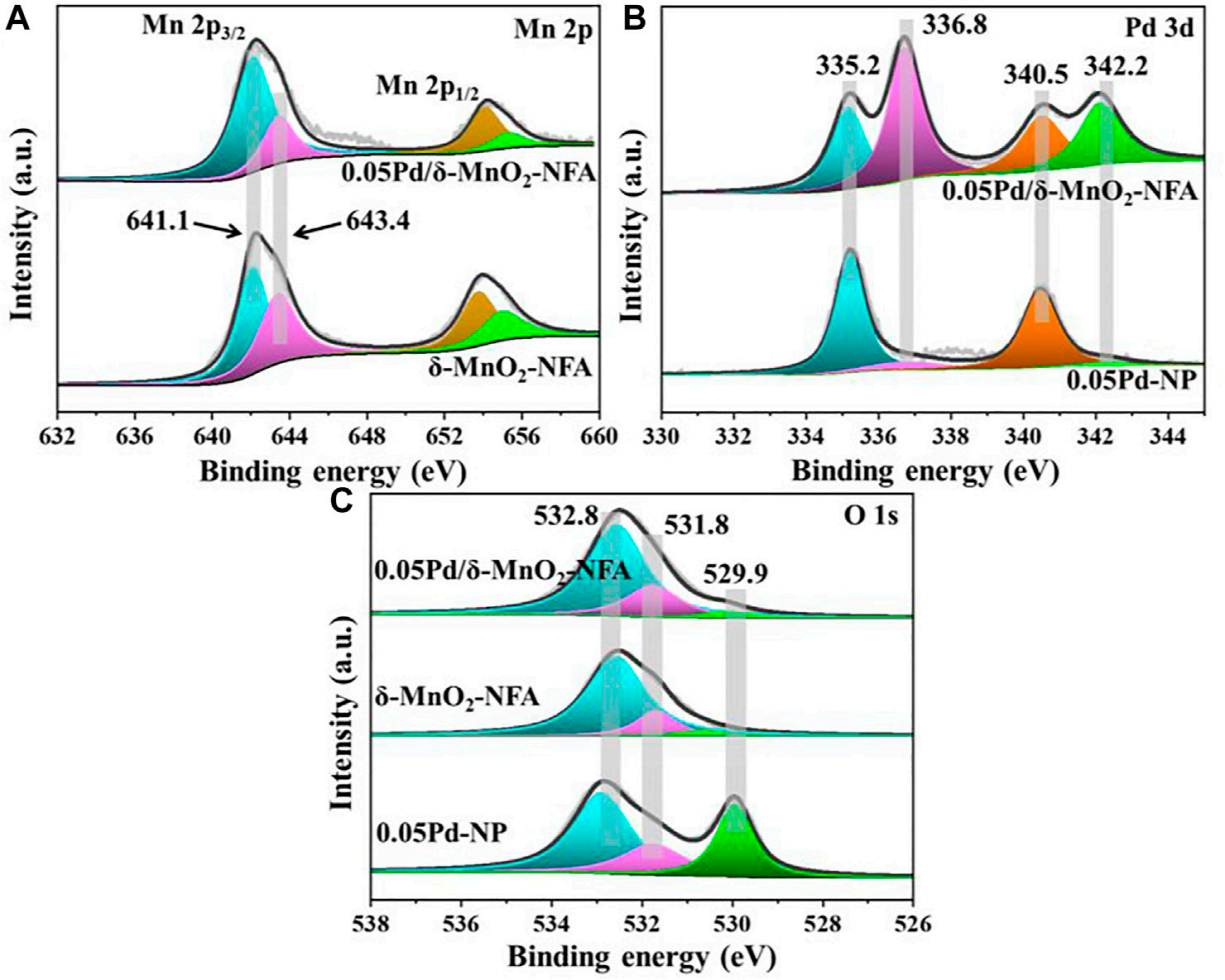
XPS spectra of 0.05Pd/δ-MnO_2_-NFA, δ-MnO_2_-NFA, and 0.05Pd-NP samples: **(A)** Mn 2p, **(B)** Pd 3d, and **(C)** O 1s.

XPS was used to determine the surface elemental properties of 0.05Pd/δ-MnO_2_-NFA, δ-MnO_2_-NFA, and 0.05Pd-NP. Figure 7A presents the Mn 2p spectra, in which two peaks at 642.3 and 654.2 eV are observed in the 0.05Pd/δ-MnO_2_-NFA and δ-MnO_2_-NFA samples, corresponding to the Mn 2p_3/2_ and Mn 2p_1/2_, respectively. Both of Mn 2p_3/2_ spectra own two components at binding energy (BE) = 642.3 and 643.4 eV, assignable to the surface Mn^3+^ and Mn^4+^ cations, respectively (Wang et al., 2020). After deposition of Pd NPs, the surface Mn^3+^/Mn^4+^ ratio rises from 1.33 (δ-MnO_2_-NFA) to 3.00 (0.05Pd/δ-MnO_2_-NFA), i.e., 0.05Pd/δ-MnO_2_-NFA owns more surface Mn^3+^ than the δ-MnO_2_-NFA (Table 3). Figure 7B illustrates the Pd 3d spectra of 0.05Pd/δ-MnO_2_-NFA and 0.05Pd-NP samples, which can be divided into four portions: the two at BE = 335.2 and 340.5 eV are belong to the surface Pd^0^ species, while the other two at BE = 336.8 and 342.2 eV are due to the surface Pd^2+^ species (Xu et al., 2017; Dong et al., 2022; Hou et al., 2022). The surface Pd^2+^/Pd^0^ ratio of 0.05Pd/δ-MnO_2_-NFA is 1.33, which is much higher than that of 0.05Pd-NP (0.15) (Table 3). From Figure 7C, there are three fixed peaks at around 529.9, 531.8, and 532.8 eV in the O 1s spectra of 0.05Pd/δ-MnO_2_-NFA, 0.05Pd-NP, and δ-MnO_2_-NFA, indicate of the surface-lattice oxygen (O_SL_) species, surface-absorbed oxygen (O_SA_) species, and hydroxyl oxygen (O_OH_) species, respectively (Yamaguchi et al., 2020; Fu et al., 2022). The O_SA_/O_SL_ ratio decreases in the order of 0.05Pd/δ-MnO_2_-NFA (0.86) > 0.05Pd-NP (0.76) > δ-MnO_2_-NFA (0.42) (Table 3), which is in accord with their catalytic performance sequence.

**TABLE 3:** Surface elemental properties of the monolithic samples.

Sample	Surface elemental properties		
	Mn^3+^/Mn^4+^ molar ratio	Pd^2+^/Pd^0^ molar ratio	O_SA_/O_SL_ molar ratio
0.05Pd/δ-MnO_2_-NFA	3.00	1.33	0.86
0.05Pd-NP	—	0.15	0.76
δ-MnO_2_-NFA	1.33	—	0.42

The XPS results testify that the strong interaction between Pd NPs and δ-MnO_2_ nanoflowers existed in the 0.05Pd/δ-MnO_2_-NFA sample. Such a strong synergy is probably because of the electron transfer proceeding at the interface between Pd and δ-MnO_2_ (Pd^0^ + 2Mn^4+^ → Pd^2+^ + 2Mn^3+^) (Xu et al., 2017), leading to the increase in the concentration of both surface Mn^3+^ and Pd^2+^ species. To maintain the electroneutrality, more oxygen vacancies should be generated on the surface of δ-MnO_2_ nanoflowers because of the incremental surface Mn^3+^ concentration; this result is conducive to activating the O_2_ to form larger amount of O_SA_ species (Liu et al., 2017; Dong et al., 2022). As is well known, abundant O_SA_ species can significantly facilitate the reaction process of toluene oxidation and further boost the catalytic performance (Xie et al., 2018; Shen et al., 2020). Therefore, the 0.05Pd/δ-MnO_2_-NFA with the most abundant O_SA_ species exhibits the optimal catalytic ability toward toluene removal.

In addition to the surface structural properties, the size of Pd NPs is another pivotal factor that directly affects the catalytic activities for toluene combustion. As we known, the Pd NPs with relatively small size can bring about high ratio of surface to volume and supply additional active sites for the adsorption of O_2_ and toluene, thereby enhancing the reactivity in toluene combustion (Li et al., 2017b; Lou et al., 2020b; Song et al., 2020; Cai et al., 2022). As discussed above, the optimum catalytic performance of 0.05Pd/δ-MnO_2_-NFA is closely related to the strong interaction between Pd NPs and δ-MnO_2_ nanoflowers, abundant O_SA_ species, and small size of Pd NPs.

### Possible reaction mechanism

**FIGURE 8:**
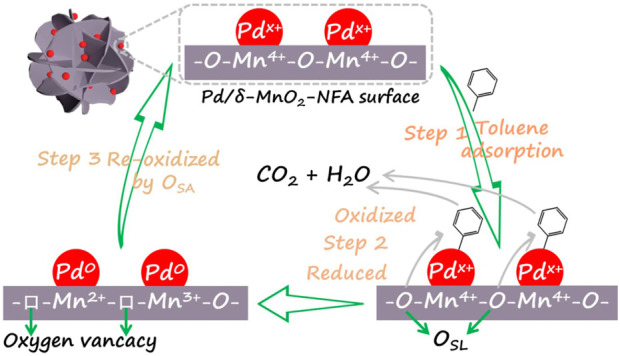
Possible reaction mechanism for toluene oxidation over 0.05Pd/δ-MnO_2_-NFA sample.

It has been reported that benzene series (e.g., toluene and *o*-xylene) on the Pd-supported catalysts is based on the Mars-van Krevelen mechanism (Bi et al., 2020; Song et al., 2020; Hyok Ri et al., 2021). Taking toluene combustion for example, the reaction pathway of toluene complete combustion for the 0.05Pd/δ-MnO_2_-NFA sample is depicted in Figure 8, which includes three main steps. (Step 1) Toluene molecule is absorbed onto the monolithic catalyst *via* the Pd^x+^ species (Pd^2+^ and Pd^0^). (Step 2) The adsorbed toluene is activated and oxidized by O_SL_ from the 0.05Pd/δ-MnO_2_-NFA surface, which then completely converts into CO_2_ and H_2_O. Meanwhile, the surface of 0.05Pd/δ-MnO_2_-NFA is reduced, thus lowing the valence of surface Mn^4+^ and Pd^2+^ ions and generating the surface oxygen vacancies. (Step 3) The reduced surface of 0.05Pd/δ-MnO_2_-NFA is timely re-oxidized by O_SA_ supplied from gaseous O_2_.

## Conclusion

In this work, honeycomb cordierite coated with uniform δ-MnO_2_ nanoflower arrays was prepared by a facile hydrothermal method, followed by loading Pd NPs onto the δ-MnO_2_ nanoflower *via* an electroless plating route. The uniformly flower-like and open structure effectively prevented the Pd NPs from further aggregation during the preparation process. Owing to the strong interaction between Pd NPs and δ-MnO_2_ nanoflowers, plentiful O_SA_ species, and small size of Pd NPs, the resulting 0.05Pd/δ-MnO_2_-NFA sample delivered a superior catalytic activity for toluene (*T*_10_ = 149°C, *T*_50_ = 206°C, and *T*_90_ = 221°C) and *o*-xylene (*T*_10_ = 183°C, *T*_50_ = 196°C, and *T*_90_ = 206°C) combustion at a GHSV of 10,000 h^−1^, respectively. Furthermore, the 0.05Pd/δ-MnO_2_-NFA sample demonstrated the stable activity in a 30 h long-term test. Our work affords useful guidance for rational design of monolithic integrated catalysts toward high-efficiency VOCs elimination.

## Data Availability

The original contributions presented in the study are included in the article/Supplementary Material, further inquiries can be directed to the corresponding author.

## Supplementary material

Supplementary Figure S1
**(A)** Effect of Pd loading (from 0.01 to 0.07 wt%) on the toluene conversion for the Pd/δ-MnO_2_-NFA samples; **(B)** *T*_10_. *T*_50_, and *T*_90_ of the Pd/δ-MnO_2_-NFA samples with different Pd loading amounts.

Supplementary Figure S2
*O*-xylene conversion over all the monolithic samples.

Supplementary Figure S3 XRD patterns of cordierite substrate and 0.05Pd-NP.

Supplementary Figure S4
**(A)** Low- and **(B)** high-magnification FESEM images of pure cordierite substrate.

Supplementary Figure S5
**(A)** Low-magnification, **(B)** high-magnification, and **(C)** side-view FESEM images of δ-MnO_2_-NFA.

Supplementary Figure S6 Nitrogen adsorption-desorption isotherms of δ-MnO_2_-NFA and pure cordierite substrate.
