# Supplementary material for: Pd/δ-MnO2 nanoflower arrays cordierite monolithic catalyst toward toluene and o-xylene combustion
Source: Front Chem. 2022 Oct 13;10:978428. doi: 10.3389/fchem.2022.978428 (PMC9606343; doi:10.3389/fchem.2022.978428)
Supplement: Supplementary file 1 [file DataSheet1.docx]

**Supplementary Material**


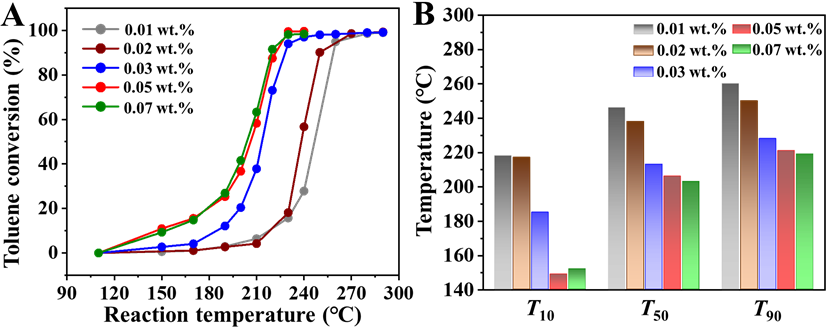


**FIGURE S1 (A)** Effect of Pd loading (from 0.01 to 0.07 wt.%) on the toluene conversion for the Pd/δ-MnO_2_-NFA samples; **(B)** *T*_10_. *T*_50_, and *T*_90_ of the Pd/δ-MnO_2_-NFA samples with different Pd loading amounts.


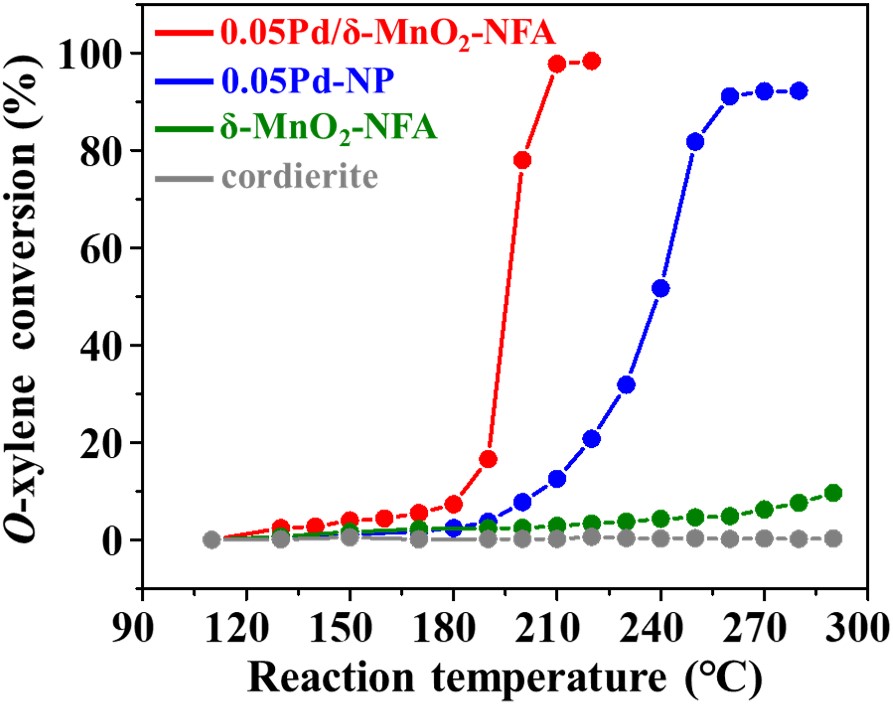


**FIGURE S2** *O*-xylene conversion over all the monolithic samples.


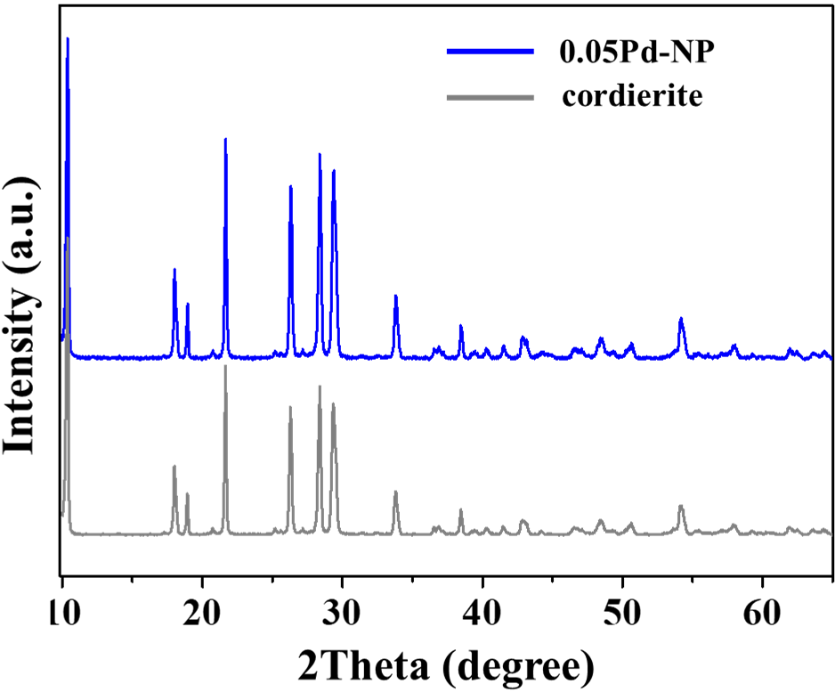


**FIGURE S3** XRD patterns of cordierite substrate and 0.05Pd-NP.


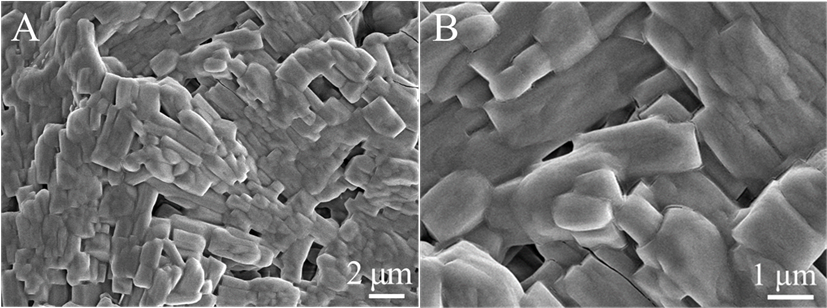


**FIGURE S4 (A)** Low- and **(B)** high- magnification FESEM images of pure cordierite substrate.


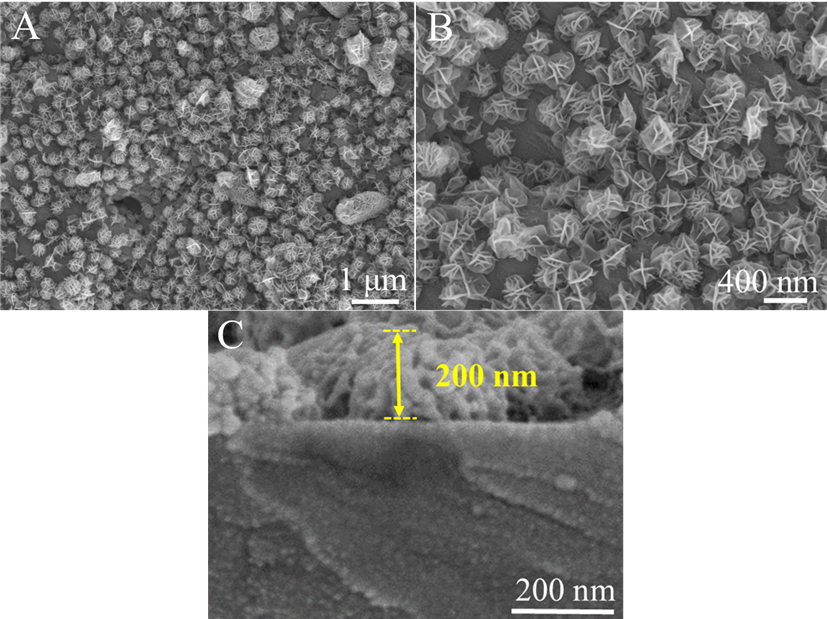


**FIGURE S5 (A)** Low-magnification, **(B)** high-magnification, and **(C)** side-view FESEM images of δ-MnO_2_-NFA.


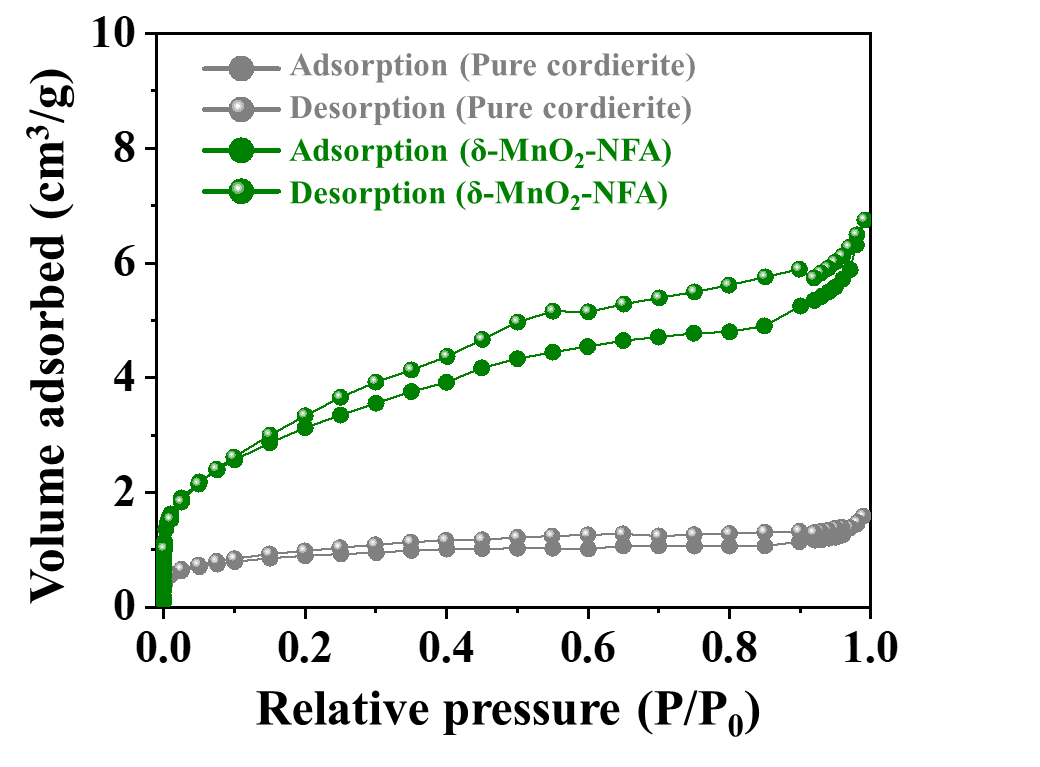


**FIGURE S6** Nitrogen adsorption-desorption isotherms of δ-MnO_2_-NFA and pure cordierite substrate.
